# Elevated CO_2_ Has Little Influence on the Bacterial Communities Associated With the pH-Tolerant Coral, Massive *Porites* spp.

**DOI:** 10.3389/fmicb.2018.02621

**Published:** 2018-11-01

**Authors:** Paul A. O’Brien, Hillary A. Smith, Stewart Fallon, Katharina Fabricius, Bette L. Willis, Kathleen M. Morrow, David G. Bourne

**Affiliations:** ^1^College of Science and Engineering, James Cook University, Townsville, QLD, Australia; ^2^Australian Institute of Marine Science, Townsville, QLD, Australia; ^3^AIMS@JCU, Townsville, QLD, Australia; ^4^ARC Centre of Excellence for Coral Reef Studies, James Cook University, Townsville, QLD, Australia; ^5^Research School of Earth Sciences, The Australian National University, Canberra, ACT, Australia; ^6^Department of Environmental Science and Policy, George Mason University, Fairfax, VA, United States

**Keywords:** ocean acidification, microbiome, coral, volcanic seep, *Porites*

## Abstract

Ocean acidification (OA) as a result of increased anthropogenic CO_2_ input into the atmosphere carries consequences for all ocean life. Low pH can cause a shift in coral-associated microbial communities of *p*CO_2_-sensitive corals, however, it remains unknown whether the microbial community is also influenced in corals known to be more tolerant to high *p*CO_2_/low pH. This study profiles the bacterial communities associated with the tissues of the *p*CO_2_-tolerant coral, massive *Porites* spp., from two natural CO_2_ seep sites in Papua New Guinea. Amplicon sequencing of the hypervariable V3-V4 regions of the 16S rRNA gene revealed that microbial communities remained stable across CO_2_ seep sites (pH = 7.44–7.85) and adjacent control sites (ambient pH = 8.0–8.1). Microbial communities were more significantly influenced by reef location than pH, with the relative abundance of dominant microbial taxa differing between reefs. These results directly contrast with previous findings that increased CO_2_ has a strong effect on structuring microbial communities. The stable structure of microbial communities associated with the tissues of massive *Porites* spp. under high *p*CO_2_/low pH conditions confirms a high degree of tolerance by the whole *Porites* holobiont to OA, and suggest that pH tolerant corals such as *Porites* may dominate reef assemblages in an increasingly acidic ocean.

## Introduction

Increasing levels of atmospheric carbon dioxide from anthropogenic emissions have led to higher CO_2_ concentrations in the surface seawaters of the oceans, changing seawater chemistry (Caldeira and Wickett, 2003; Doney et al., 2009). The chemical reaction between CO_2_ and water releases hydrogen ions (H^+^), thereby reducing pH (Doney et al., 2009). Seawater pH is defined by the negative base-10 logarithm of H^+^, thus a near 30% increase in H^+^ since the industrial revolution equates to a decrease of 0.1 pH units in global surface seawater (Rhein et al., 2013). Consequently, over the last 200 years, the average pH of global surface seawater has been reduced by ∼0.1 units to ∼8.1, and based on current CO_2_ emissions modeling, pH is predicted to decrease by a further 0.2–0.4 units by 2100. This decrease will lead to changes in ocean chemistry not seen for the past 23 million years (Rhein et al., 2013). Although seawater pH fluctuates (especially on coral reefs) with season, depth and productivity (Joint et al., 2011), the reduction in baseline pH levels, termed ocean acidification (OA), represents a growing concern for global ocean ecosystems (O’Brien et al., 2016).

Coral reefs have been a focal point for understanding the effects of OA, as skeletal growth (i.e., calcification) of scleractinian (reef-building) corals is progressively impeded at low pH (Gattuso et al., 1998). Reduced calcification in reef-building species poses a serious long-term threat to the future of coral reefs globally. A coral colony represents a meta-organism, a complex and interdependent association between a coral animal, endosymbiotic dinoflagellates of the family *Symbiodiniaceae* (LaJeunesse et al., 2018), and diverse members of the Bacteria, Archaea, Fungi, Protista, Apicomplexa and viruses (Rohwer et al., 2002; Bourne et al., 2016). Members of this inter-kingdom partnership are collectively referred to as the coral holobiont. The roles of bacteria within complex partnerships are becoming apparent, and it is now widely accepted that microbes in general are critical to the health and survival of multicellular organisms such as the coral holobiont (Rosenberg et al., 2007; McFall-Ngai et al., 2013).

Bacteria perform essential functions for the health of the coral host. For example, nitrogen-fixing bacteria in coral tissues are believed to help sustain the coral*-Symbiodiniaceae* relationship (Lesser et al., 2004; Lema et al., 2012), while carbon-fixing bacteria may provide an additional energy source when *Symbiodiniaceae* densities are low (Fine and Loya, 2002; Rosenberg et al., 2007). Antibiotic properties of bacteria within coral mucus are important for structuring coral-associated microbial communities, ensuring habitat niches for beneficial bacteria and preventing invasion by potentially opportunistic or pathogenic microorganisms (Ritchie, 2006). Metabolism of dimethylsulfoniopropionate (DMSP), which is produced by both the coral host and *Symbiodiniaceae*, provides antioxidants that protect coral tissues against harmful reactive oxygen species (Sunda et al., 2002; Raina et al., 2013). Under ideal conditions, microorganisms form communities that benefit the host animal through nutrient cycling and disease prevention. However, stressful environmental conditions can alter microbial community composition, which can negatively affect the fitness and survival of the holobiont.

Disruptions to microbial community composition and function are thought to adversely affect coral host fitness, and have been documented in response to a variety of environmental stressors, including temperature, eutrophication, pollution, and pH (Kline et al., 2006; Bourne et al., 2008; Webster et al., 2013, 2016; Ziegler et al., 2016). In general, perturbations resulting in dysbiotic community states are stochastic rather than deterministic, resulting in a variable and unstable microbiome (the aptly named Anna Karenina principle) (Zaneveld et al., 2017). In response to OA, aquaria-based studies have demonstrated that coral microbial communities generally shift toward an unhealthy state in response to low pH (Thurber et al., 2009; Meron et al., 2011). However, research has primarily focused on coral species that are sensitive to changes in pH (Fabricius et al., 2011; Strahl et al., 2015), and/or involve pH levels that are beyond what is predicted for the coming century (Thurber et al., 2009). Studies conducted *in situ,* where pH levels are more representative of future conditions, have produced variable results. For example, no change in coral bacterial communities was detected following translocation of two Mediterranean coral species along a natural pH gradient in the Gulf of Naples (Meron et al., 2012). Likewise, the endolithic community associated with massive *Porites* spp. did not change significantly between ambient and low pH sites found at naturally occurring CO_2_ seeps in Papua New Guinea (PNG) (Marcelino et al., 2017). Conversely, large shifts in the structure of tissue-associated bacterial communities were found in the corals *Acropora millepora* and *Porites cylindrica* across the same CO_2_ seep in PNG (Morrow et al., 2014).

Natural volcanic CO_2_ seeps have been used as experimental settings to assess the long-term effects of OA on marine organisms *in situ* due to their naturally high *p*CO_2_ levels (Hall-Spencer et al., 2008; Fabricius et al., 2011, 2014; Morrow et al., 2014). Within the D’Entrecasteaux Islands of the Milne Bay province of PNG, coral reefs are found within volcanic seeps that release 99% pure CO_2_ into the surrounding seawater. At these seeps, median pH levels range from 7.28–8.01 (Fabricius et al., 2014) and are within the range of “business-as-usual” predictions for the coming century (Rhein et al., 2013). Benthic invertebrate community structure, abundance and diversity are dramatically different at these seeps compared with nearby control sites exposed to the same temperatures, currents, geomorphology and light levels (Fabricius et al., 2011, 2014; Morrow et al., 2014). While the mechanism driving survival of communities at high *p*CO_2_ sites is unclear, evidence suggests that host-associated microbial communities resilient to low pH enable host fitness and survival (Morrow et al., 2014).

Some coral species, such as species of the genus *Porites* with massive morphology, appear tolerant to high *p*CO_2_ and are present in high abundance within the seep environments (Fabricius et al., 2011). While this coral maintains its physiological performance under low pH conditions (Edmunds et al., 2012; Strahl et al., 2015), and contains a stable endolithic community (Marcelino et al., 2017), it remains unknown whether microbial communities associated with the tissues of this *p*CO_2_-tolerant coral are impacted by low pH conditions. This study profiled the bacterial communities of massive *Porites* spp. at two CO_2_ seeps in PNG and compared microbial community profiles to corals from adjacent control sites. Through this comparison, we determined that these *p*CO_2_-tolerant corals can maintain more stable microbial communities than their *p*CO_2_-sensitive counterparts.

## Materials and Methods

### Study Site and Sample Collection

**FIGURE 1:**
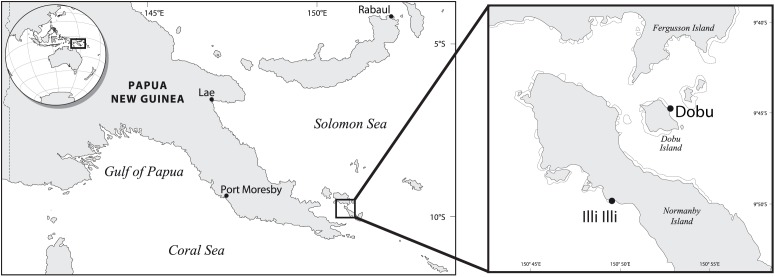
Study site locations in Papua New Guinea (adapted from Fabricius et al., 2011).

A total of 15 samples (*n* = 3 per site) were collected at two natural volcanic CO_2_ seep sites; Illi Illi near Upa-Upasina (herewith Illi), Normanby Island, and Dobu, Dobu Island, in the D’Entrecasteaux Islands, Milne Bay Province, Papua New Guinea (Figure 1). Seeps at both Illi (9.7625S, 150.8186E) and Dobu (9.7366S, 150.8691E) expel 99% pure CO_2_ into the water column, resulting in median ambient pH levels of 7.77 and 7.73, respectively. These pH levels are consistent with predictions for ocean chemistry over the next century. Control sites were less than 500 m away and have similar seawater temperature and salinity (Fabricius et al., 2011, 2014), with median ambient pH levels of 7.97 and 8.02 at Illi and Dobu, respectively (Fabricius et al., 2011).

Coral samples were collected on SCUBA at a depth of 3–5 m using a hammer and chisel in November 2014. Samples were collected from 3 colonies of massive *Porites* spp. at each of the two seeps and corresponding control sites, and at a second control site at Dobu. Samples were brought to the surface and briefly kept in running seawater (1.0 μm filtered) until being processed (<2 h). Coral tissues were rinsed with 0.22 μm filtered seawater and then removed from the skeleton using pressurized air (∼130–150 PSI) from a blow gun with sterile tips into a sterile bag (Whirlpak^®^, Nasco). Tissue blastate (2 mL) was pelleted in a fixed-angle centrifuge (20,000 × *g* for 20 min at 4°C; Beckman-Coulter, Brea, CA, United States), seawater supernatant decanted, tissue pellets snap frozen in liquid nitrogen, and stored at −80°C.

### Coral Cover and Diversity

Coral cover and diversity were estimated at each site by placing five replicate 50 × 2 m belt transects parallel to shore at a depth of 3–7 m. A total of 25 photographs of 1 × 1 m quadrats were taken on alternating sides of the transect every 2 m along each transect. Photographs were analyzed using photoQuad (Trygonis and Sini, 2012) by overlaying 50 random points over the image and identifying the underlying coral to genus. Shannon’s diversity index (H) was calculated based on the number of coral genera for each transect. To determine if coral cover, the cover of massive *Porites*, and diversity varied significantly between seep and control sites, a generalized linear mixed effect model was performed using the package MASS (Venables and Ripley, 2002) in R Studio (R Core Team, 2016). The model nested seeps within reefs, and had transects as random factors, and a Poisson distribution was used for the percentage cover data.

### Sample Processing and Sequencing of 16S rRNA Gene

Samples were thawed at 4°C and DNA was extracted using the PowerSoil DNA Isolation Kit, following the manufacturer’s instructions (MoBio Laboratories, Inc; Carlsbad, CA, United States), except a 10 min 65°C heating step was added prior to bead beating. Sequencing was completed at the Molecular Research DNA laboratory (MR DNA; Shallowater, TX, United States). The hypervariable V3-V4 regions were targeted using the 16S rRNA gene primer set, 341F (CCTACGGGNGGCWGCAG) and 785R (GACTACHVGGGTATCTAATCC) (Klindworth et al., 2013), in a 30 cycle PCR using the HotStarTaq Plus Master Mix Kit (Qiagen, United States) under the following conditions: 94°C for 3 min, 28 cycles of 94°C for 30 s, 53°C for 40 s and 72°C for 1 min, followed by a final elongation at 72°C for 5 min. After amplification, samples were pooled in equal proportions based on their molecular weight and DNA concentrations. Pooled samples were purified using calibrated Ampure XP beads and then used to prepare a DNA library by following the Illumina TruSeq DNA library preparation protocol. Sequencing was performed on the Illumina MiSeq platform (2 × 300 bp) following the manufacturer’s guidelines.

### 16S rRNA Gene Amplicon Analysis

Sequence reads of all samples were processed using QIIME2 (Caporaso et al., 2010). In summary, reads were demultiplexed and barcodes and primer regions were removed. Reads were filtered for quality and chimeric sequences using dada2 (Callahan et al., 2016). Taxonomic classification was assigned using a naïve Bayes classifier trained on the extracted region of interest from the SILVA 16S rRNA reference alignment [Release 132 (Quast et al., 2013)]. All sequences classified as chloroplast, mitochondria, or eukaryota were removed. The resulting amplicon sequence variant (ASV) table was used for statistical analysis in R Studio (R Core Team, 2016) using the packages vegan, car, VennDiagram, pheatmap, and IndicSpecies (De Caceres and Legendre, 2009; Chen and Boutros, 2011; Fox and Weisberg, 2011; Kolde, 2015; Oksanen et al., 2016).

The percent relative abundance of each microbial phylum present in each sample was calculated, and the top ten most abundant taxa were plotted. All additional analyses were conducted at the genus level. Alpha diversity was calculated using Shannon’s diversity index. Sequence count data were standardized with a Wisconsin double-standardization [i.e., each value is standardized by the column maximum followed by the row total (Bray and Curtis, 1957)]. Patterns in microbial community composition were visualized using non-parametric multidimensional scaling (NMDS) based on Bray–Curtis dissimilarity. Permutational multivariate analysis of variance (PERMANOVA) was used to identify differences in microbial community diversity as a function of location and CO_2_ treatment.

Core microbial taxa were identified as the genera occurring in 100% of samples across all sites. Indicator taxa were identified for each treatment (i.e., seep and control) and each location (i.e., Dobu and Illi) using the R package IndicSpecies (De Caceres and Legendre, 2009). Each microbial taxon was tested for significant correlation using 999 permutations. Sequence counts for the resulting indicator species were standardized to the number of total sequences per sample, and heatmaps were created to visualize relative abundance.

### Modeling pH Using ^14^C

We utilized the ^14^C content in both the control and seep corals to estimate the seawater pH. Approximately 7 mg of coral powder was weighed into Vacutainer^TM^ blood vials. The samples were acidified under vacuum with 0.3 ml of 85% orthophosphoric acid at 70°C to evolve CO_2_ gas. The CO_2_ gas was cryogenically purified and converted to graphite using H_2_/Fe (Vogel et al., 1984). The ^14^C of the resulting graphite was measured on a Single Stage Accelerator Mass Spectrometer at the Australian National University (Fallon et al., 2010). Analytical results are reported as F^14^C using the Oxalic Acid-I, corrected for d^13^C and background subtracted using Carrera Marble (Supplementary Table S1 and Supplementary Equations). The pH values for seawater were modeled using CO2SYS (Pierrot et al., 2006) from *in situ* alkalinity and salinity measurements, high resolution IGOSS satellite temperature records and the calculated impacted dissolved inorganic carbon (DIC).

## Results

### Coral Cover and Diversity

**FIGURE 2:**
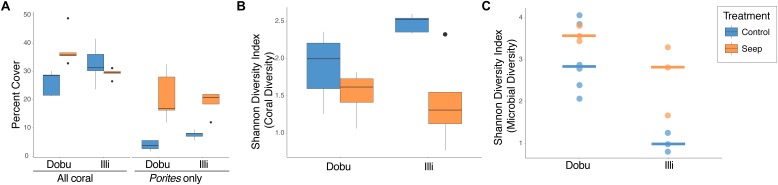
Percent total coral cover and massive *Porites spp.* cover **(A)**; Shannon’s diversity index for scleractinian corals **(B)**; and Shannon’s diversity index for microbial communities associated with massive *Porites spp.* **(C)** at control and seep sites at Dobu and Illi reefs.

There was no significant difference in total coral cover between seep and control sites (GLM; *x*^2^ = 2.6_(1)_, *p* = 0.11; Figure 2A and Supplementary Figure S3). However, there was an increase in coral cover at the Dobu seep (37.93%) compared to the Dobu control sites (26.06%), largely due to a fivefold increase in cover of massive *Porites spp.,* from 4.13% at the Dobu control site to 21.41% at Dobu seep. At Illi, percent cover of massive *Porites* spp. also increased from 7.89% cover at the control site to 19.28% at the seep. Across both reefs, percent cover of massive *Porites* spp. was significantly higher at the seep compared to the control sites (GLMM; *x*^2^ = 49.0_(1)_, *p* < 0.001; Figure 2A). As a result, coral community diversity was significantly lower at the seep compared to the control sites at both reefs (ANOVA, *F*_(2,17)_ = 7.356, *p* = 0.005; Figure 2B).

### Bacteria Community Diversity Comparisons

**FIGURE 3:**
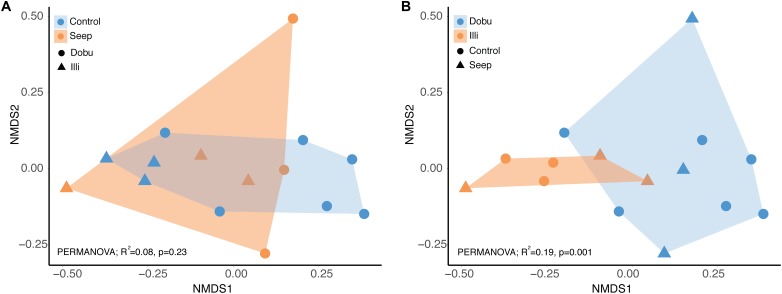
Microbial community composition visualized using NMDS. Two colorations of the same plot are shown to visualize the effect of treatment **(A)** or reef **(B)**.

A total of 875,140 sequence reads were recovered from all 15 samples, though following filtering, only 119,332 high quality reads were subsequently used for taxonomic classification. A total of 537 bacterial genera (comprised of a total 683 sequence variants) were detected across all samples. Rarefaction analysis confirmed sampling depth was sufficient to estimate the total diversity of each sample, with species detection curves reaching an asymptote after a sequencing depth of approximately 450 reads (Supplementary Figure S1). Shannon’s diversity index of microbial communities associated with massive *Porites spp.* showed a significant interaction between locations and treatments (ANOVA; *F*_(3,11)_ = 8.991, *p* = 0.003). Interestingly, mean alpha diversity was significantly lower at the Illi control reef (1.03 ± 0.13 SE) than the Illi seep (2.61 ± 0.48 SE; Figure 2C). At Dobu, microbial diversity was similar at control and seep sites (3.02 ± 0.32 and 3.62 ± 0.11, respectively; Figure 2C). Across all samples, community composition was significantly different between the two reefs (PERMANOVA; *R*^2^ = 0.19, *p* = 0.001; Figure 3A), but there was no significant difference between seep and control sites (PERMANOVA; *R*^2^ = 0.08, *p* = 0.23; Figure 3B). These results suggest that the greatest influence on massive *Porites* spp. microbial communities was the location of the reef, while the effect of higher *p*CO_2_ at the seeps had little influence at either reef. The samples from Dobu were not tightly clustered, suggesting a high degree of variability in microbial community composition (Figure 3). Conversely, the Illi samples were more tightly clustered, suggesting greater similarity in community composition.

**FIGURE 4:**
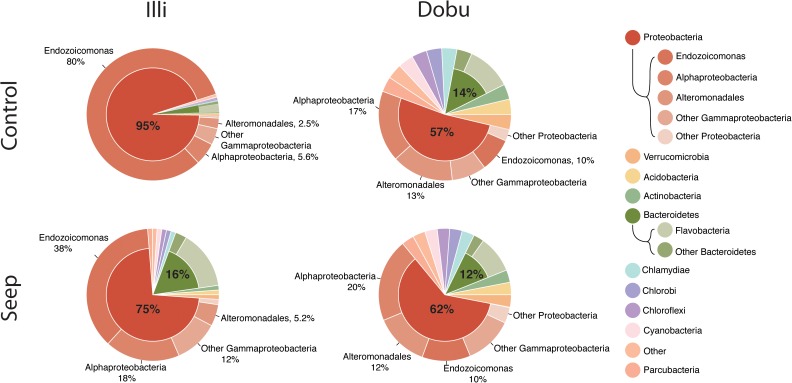
Ten most abundant microbial phyla associated with massive *Porites. “*Other” represents all microbes not in the top 10. The two most abundant phyla, *Proteobacteria* and *Bacteroidetes*, are split further into classes. *Gammaproteobacteria* are split further to illustrate the high relative abundance of the genus *Endozoicomonas* (outside ring). Percentages are mean proportion of total sequences.

**FIGURE 5:**
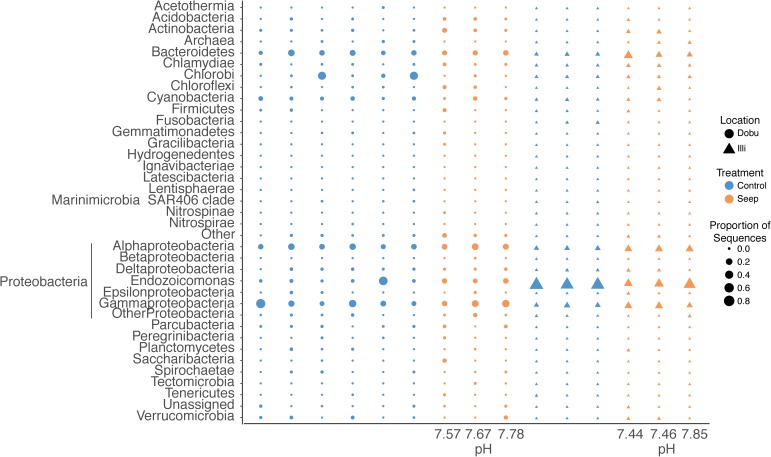
Relative abundance of the 37 microbial phyla associated with massive *Porites spp.* at seep and control sites at Illi and Dobu reefs. *“*Other” represents all that could not be classified below Kingdom level. “Archaea” represents all sequences affiliated to this domain. “Unassigned” represents all sequences that could not be assigned taxonomy. Sequences affiliated with *Proteobacteria* are split into classes (*Alphaproteobacteria, Betaproteobacteria, Deltaproteobacteria, Epsilonproteobacteria, and Gammaproteobacteria*) and genus (*Endozoicomonas*) to display higher resolution of dominant taxa associated with this phylum. Values below each column represent the pH value of each coral skeleton collected from the seep sites as analyzed by ^14^C chemistry.

A total of 37 microbial phyla were identified within the 16S rRNA dataset. Sequences affiliated with the phylum *Proteobacteria* were the most abundant across all samples at both the seep and control sites, representing >93% of sequences from the Illi control site and between 29 and 82% of sequences from the Dobu control site (mean 94.7 ± 0.6 and 56.9 ± 9.6%, respectively; Figures 4, 5 and Supplementary Table S2). Two replicate samples from the Dobu control site with lower relative abundance of *Proteobacteria* displayed higher abundance (up to 45%) of sequences from the phylum *Chlorobi* (i.e., green sulfur bacteria; Figure 5). In general, both Illi and Dobu seep sites showed a lower abundance of *Proteobacteria* compared to the Illi control reef (Figures 4, 5). No trend was observed in the relative abundance of *Proteobacteria* between the Dobu control and seep sites (Figures 4, 5).

Within the *Proteobacteria*, sequences associated with the genus *Endozoicomonas* and family *Alteromonadaceae* (class *Gammaproteobacteria*) and class *Alphaproteobacteria* made up the vast majority in all samples. At the Illi control site, all 3 samples were dominated by members affiliated with *Endozoicomonas*, comprising an average of 79.6 ± 2.9% of all taxa present (Figures 4, 5 and Supplementary Table S2). Members of the *Endozoicomonas* were also the dominant taxa at the Illi seep, however, their abundance was lower at 38.3 ± 10.2% of all sequences. At Dobu, the relative abundance of *Endozoicomonas* sequences was reduced compared to Illi (Figures 4, 5), but was similar across seep and control sites (10.4 ± 1.4 and 10.1 ± 7.7% of sequences, respectively).

Where relative abundance of *Endozoicomonas* was low, the relative abundance of sequences affiliated with the order *Alteromonadales* was generally higher. The Dobu control sites contained the lowest relative abundance of *Endozoicomonas*, and subsequently the greatest relative abundance of sequences related to *Alteromonadales*, with a mean of 13.4 ± 4.8% (ranging from 3–35.6%), compared to 11.6 ± 4.2% at the seep. Conversely, the seep site comprised slightly more sequences related to *Alphaproteobacteria*, with a mean of 20.4 ± 3%, compared to 17 ± 2.3% at the control. The Illi seep site contained a higher relative abundance of *Alphaproteobacteria* than the control, with a mean of 18.3 ± 0.4% compared to 5.6 ± 0.5%, respectively. *Alteromonadales* were present at low abundances in both Illi control and seep samples, with a mean of 2.5 ± 0.2 and 5.2 ± 1.2%, respectively. Overall, *Alteromonadales* and *Alphaproteobacteria* were present in marginally higher relative abundance at Dobu than Illi, regardless of treatment (Figure 4), suggesting that patterns in relative abundance are more strongly influenced by reef than by pH.

### Patterns in the Core Microbiome and Indicator Species

**FIGURE 6:**
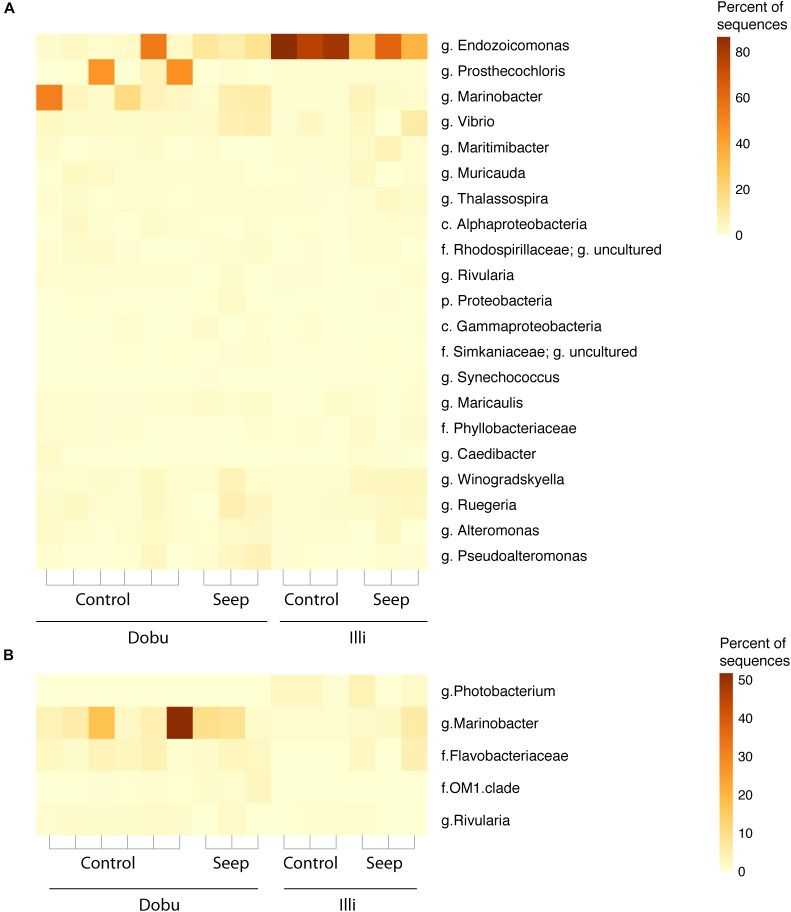
**(A)** Relative proportion of core microbes (the 22 taxa found in 100% of samples). **(B)** Relative proportion of indicator taxa (taxa found to be significantly correlated (*p* < 0.05) to each site). Taxa are identified to the lowest possible classification, where p. is phylum, c. is class, f. is family, and g. is genus.

The core microbiome and indicator taxa were investigated to determine if the high *p*CO_2_ environment at natural seeps may influence the presence of potentially beneficial bacterial taxa. Across all sites, 22 taxa (66,139 sequences) were present in every sample (Figure 6 and Supplementary Figure S2). The majority of core taxa were present in low abundances across all samples, with the genus *Endozoicomonas* being the most abundant, accounting for 33,974 sequences (51.4%), followed by *Prosthecochloris spp.* (class *Chlorobia*; 13.5%*)* and *Marinobacteria spp.* (order *Alteromonadales;* 9.6%). No trend in relative abundance was observed between seep and control sites across both reefs.

Dobu *Porites* spp. supported three indicator taxa: the genus *Marinobacter* (*p* = 0.009), the family *Flavobacteriaceae* (*p* = 0.008), and the family OM1 clade of *Acidimicrobiales* (*p* = 0.016). The genus *Marinobacter* was the most abundant indicator species, representing a mean of 11.6% of sequences in Dobu samples. Only one taxon was found to be significantly indicative of Illi *Porites* spp.: the genus *Photobacterium* (c. *Gammaproteobacteria,* f. *Vibrionaceae; p* = 0.005*)* was present at low but consistent abundances, representing a mean of 1.8% of sequences from all Illi samples (Figure 6). The genus *Rivularia* was indicative of control sites (*p* = 0.013), but was present in very low abundances (mean of 0.9% of all control sample sequences). Notably, no indicator taxa were detected for seep sites.

### pH Values Modeled Using ^14^C

A precise measurement of seawater pH was given for each coral living in the seep environment (Supplementary Table S1). Seawater pH was variable among colonies and dropped below what is predicted for the coming century. At the Dobu seep, pH varied from 7.67 to 7.78, while at the Illi seep pH varied from 7.44 to 7.85.

## Discussion

The rising partial pressure of carbon dioxide (*p*CO_2_) in the oceans and subsequent OA poses a serious threat to marine life, particularly to calcifying organisms such as reef-building corals. However, corals show varying degrees of sensitivity to high *p*CO_2_ and some may even benefit from the change in ocean chemistry (Morrow et al., 2014; Strahl et al., 2015; Strahl et al., 2016). In the current study, we show that massive *Porites* corals that occur in high abundance at low pH/high *p*CO_2_ seep sites in PNG host robust bacterial communities that are stable across a broad range of environmental pH levels.

### Massive *Porites* spp. Dominate CO_2_ Seeps in Papua New Guinea

The high abundance of massive *Porites* spp. at the PNG seeps (pH 7.44–7.85) in comparison to adjacent control sites demonstrates that *Porites spp.* may be able to withstand lower pH conditions than those predicted for the coming century (i.e., ∼7.8) (Rhein et al., 2013). The dominance of massive *Porites spp.* at the seeps led to a significant decrease in coral diversity in comparison to control sites. These findings are consistent with previous reports of coral community assemblages at the CO_2_ seeps of the D’Entrecasteaux Islands, in which structurally complex branching and tabular corals were found to dominate control sites, while massive, boulder formations dominated at the seeps (Fabricius et al., 2011; Morrow et al., 2014). Community changes resulting in a loss of topographical complexity have important impacts on the reef ecosystem as many coral reef organisms seek refuge in the complex habitat provided by branching and tabular coral species (Graham and Nash, 2013; Fabricius et al., 2014). Therefore, it is critical to understand what drives the success of massive *Porites* in low pH/high *p*CO_2_ environments. Evidence suggests that ecological success is linked to both physiological (Wall et al., 2016) and microbial mechanisms (Marcelino et al., 2017), with our data supporting the concept that *Porites* maintains a stable microbial community, even under extreme *p*CO_2_ conditions.

### Response of Massive *Porites* spp. Microbial Communities to Low pH/High *p*CO_2_

The microbial communities of massive *Porites* were highly similar between colonies in volcanic seep habitats (pH 7.44–7.85) and nearby colonies living under ambient pH conditions (pH ∼8.1). With the exception of the high abundance of *Endozoicomonas* at the Illi control site, there were negligible differences in the relative abundance of dominant taxa between control and seep colonies, suggesting that the microbiome of *Porites* spp. is stable across *p*CO_2_ conditions. The major difference in microbial community was between the two reefs (i.e., Illi versus Dobu), and was largely characterized by increases in the abundance of *Endozoicomonas* and decreases in *Chlorobi* at Illi reef. Previous work investigating the endolithic community of *Porites* spp. at the PNG seeps found no significant difference in community composition between seep and control sites (Marcelino et al., 2017), suggesting the skeletal microbial community is robust to low pH conditions. Our results align with this previous work, demonstrating that the tissue-associated microbial community is also robust to high *p*CO_2_ conditions. Our findings that massive *Porites* show little change in microbial community structure across pH levels strongly contrasts with previous findings focused on *p*CO_2_-sensitive corals (Morrow et al., 2014). For instance, it was previously demonstrated that a significant decline in the abundance of the coral species *A. millepora* and *P. cylindrica* at CO_2_ seeps co-occurred with a substantial shift in associated microbial communities (Morrow et al., 2014). It may be hypothesized that one factor underpinning the tolerance of corals to a low pH environment could be the association with a microbial community that is robust toward changing pH levels.

An alternative hypothesis to explain the resilience of corals at seep sites is that the physiological performance of the coral host influences the diversity and stability of the associated microbial community. Recent work on massive *Porites* has shown that this coral is able to regulate and maintain its internal pH when the surrounding seawater pH is reduced to ∼7.9 (Wall et al., 2016). This method of pH regulation involves the active removal of protons (H^+^) from the calicoblastic space (Venn et al., 2013), however, it is unknown what energetic trade-offs may accompany this in massive *Porites* at the seeps. Massive *Porites* benefit from the high *p*CO_2_ environment at the PNG seeps through an increase in net photosynthesis (however, unaltered net calcification rates) in comparison to control sites (Strahl et al., 2015). In addition to providing added nutritional support, an increase in photosynthesis raises the internal pH of the coral tissues through the removal of CO_2_ (Kühl et al., 1995). Furthermore, tissue biomass, lipid, protein and tissue energy content, fatty acid content, pigment content, and oxidative stress parameters all remained unaffected by *p*CO_2_ up to 800 μatm (Strahl et al., 2015). Similarly, aquaria-based experiments have shown that calcification in massive *Porites* appears largely unaffected by increases in *p*CO_2_ (Anthony et al., 2008; Edmunds, 2011; Edmunds et al., 2012). Massive Porites have repeatedly been shown to be one of the most *p*CO_2_-tolerant coral taxa due to their ability to maintain higher internal pH values at the site of calcification than many other scleractinians (McCulloch et al., 2012). Therefore, through the combination of internal pH regulation and increased photosynthesis, massive *Porites* corals likely maintain a more stable pH, which in turn benefits the stability of their microbial assemblages.

This interpretation is consistent with previous investigations into the microbiology and physiology of the coral species *A. millepora* at the PNG CO_2_ seeps. The *p*CO_2_-sensitive species not only displayed a shift in microbial community structure at the seeps (Morrow et al., 2014), but also have significantly lower tissue biomass and lipid content in comparison to *Porites* (Strahl et al., 2015). Additionally, although daylight calcification rates and photosynthesis in *A. millepora* were unaffected, both dark and net calcification rates were significantly reduced (Strahl et al., 2015), and likely represent the coral’s inability to regulate internal pH, or a trade-off of resources away from processes such as calcification into pH regulation. It is possible that corals sensitive to changes in surrounding pH will divert resources away from key cellular metabolic processes, thereby changing the amount and composition of exudates and subsequently influencing the associated microbial consortia. Thus, the strong physiological performance of massive *Porites* at the seep potentially supports a stable microbial community, further increasing the resilience of massive *Porites* to low pH.

### Response of Microbial Communities Associated With Massive *Porites* spp. to Reef Location

Reef location had the strongest influence on microbial communities associated with massive *Porites*. While the same dominant microbial taxa were present at both sites, changes in the relative abundance led to significant changes in overall community structure. Specifically, lower relative abundances of *Endozoicomonas* at Dobu reef were replaced with increased relative abundances of *Alteromonadales* and *Chlorobi,* and were largely responsible for the differences in microbiome diversity between reefs. In particular, the Illi control samples had the lowest microbial diversity due to the high abundance of *Endozoicomonas* at this site. Further site differences were highlighted by an indicator taxa analysis that identified the less abundant *Marinobacter, Flavobacteriaceae* and *Acidimicrobiales* as characteristic of the Dobu site. These changes were clearly reflected in the NMDS plot with multivariate data being clustered by reef location rather than CO_2_ treatment. *Marinobacter* are known to degrade some organic pollutants (Huu et al., 1999) (see below), and may indicate a difference in water quality between the two sites. However, previous measurements of seawater chemistry (excluding pH differences at the seeps) were consistent between the two reefs (Fabricius et al., 2014).

Illi and Dobu reefs are known to harbor different communities of zooplankton, where species abundances and biochemical composition vary significantly between the two reefs (Smith et al., 2016). This represents a potential difference in food quality for the coral populations, which can induce changes to the physiology of both the host and its algal symbionts (Houlbrèque and Ferrier-Pagès, 2009), and potentially its prokaryotic community as well. Differences in zooplankton communities between reefs highlight the possibility of a currently unknown factor affecting microbial communities between reefs at Illi and Dobu. However, it is not uncommon for microbial communities associated with coral to show spatial variation. Patterns in reef-specific coral-associated bacterial communities have previously been observed, with the same species of coral harboring different microbial communities in different locations (Littman et al., 2009; Morrow et al., 2012). It has also been speculated that variation in microbial community structure reflects differences in *Symbiodiniaceae* populations (Littman et al., 2010; Sogin et al., 2017), which may produce variable nutrient sources for associated microbes (Olson et al., 2009). However, it has been shown that *Symbiodiniaceae* community composition of massive *Porites* (and other corals) does not change between the locations in this study, nor do they change after life-long exposure to elevated CO_2,_ (Noonan et al., 2013), and thus it is unlikely that microbial communities in the current study were affected by *Symbiodiniaceae* identity. Lastly, massive *Porites* includes multiple species that cannot be identified in the field. It is feasible that site differences in the microbial community are due to different species inhabiting different locations and future studies on this group of corals should incorporate molecular testing to resolve the taxonomy to species level. In the current study, it remains unclear what drives the differences between the two reefs microbial communities.

### Core Microbes Associated With Massive *Porites* spp.

*Endozoicomonas* was the most common core taxa, and is considered a common marine symbiont, found in high abundance across many coral genera (Bayer et al., 2013b; Neave et al., 2016; Pogoreutz et al., 2018) and other marine invertebrates (Martínez-García et al., 2007; Jensen et al., 2010; Bayer et al., 2013a). The widespread association of *Endozoicomonas* with multiple taxa suggests that the genus has coevolved with marine invertebrates through a developed symbiotic relationship, similar to the relationship between corals and *Symbiodiniaceae* (Bayer et al., 2013a,b). Recent genomic work on *Endozoicomonas* highlighted the potential for important functional roles, including the transport of organic molecules between symbiont and host and the synthesis of amino acids (Neave et al., 2017). Due to these potentially fundamental roles of *Endozoicomonas*, this genus is emerging as a core symbiont in corals and other invertebrate taxa.

Sequences affiliated with both *Marinobacter spp.* (order *Alteromonadales*) and *Prosthecochloris* spp. (class *Chlorobia)* were also conserved across all samples but were far less abundant than *Endozoicomonas.* Interestingly, bacteria from both genera are able to grow optimally under low pH conditions (relative to ambient seawater pH), however, whether or not this confers any degree of tolerance to the coral holobiont under low pH is unknown (Gorlenko, 1970; Pringault et al., 1998; Duran, 2010). Bacterial strains from the genus *Marinobacter* are known for their ability to degrade hydrophobic organic compounds including some organic pollutants (Gauthier et al., 1992; Huu et al., 1999), and has recently been isolated from a Bryozoan sample, indicating its potential association with marine invertebrates (Romanenko et al., 2005). Direct contact with marine algae is known to transfer hydrophobic organic compounds to nearby corals, thereby stimulating microbial growth and creating a pathway for opportunistic pathogens (Barott and Rohwer, 2012). However, the role of *Marinobacter* within the coral holobiont is currently unknown.

Bacteria affiliated to *Chlorobi* have previously been found within the endolithic community of coral skeletons (Marcelino and Verbruggen, 2016; Yang et al., 2016; Marcelino et al., 2017), and have been previously discovered in the skeleton of massive *Porites* collected from the same seeps as the current study (Marcelino et al., 2017). Specifically, the green sulfur bacterium *Prosthecochloris* is likely an important member of the coral holobiont due to its role in nitrogen fixation, and has been found in the tissues of *Porites lutea* (Li et al., 2014) and dominates the green endolithic layer in the coral *Isopora palifera* (Yang et al., 2016). As *Chlorobi* appear to characterize the endolithic community, it is possible that sequences found within the tissues represent endolithic contamination when sampling. Nevertheless, it appears to be a recurrent member of the massive *Porites* holobiont community.

## Conclusion

The conditions offered by natural CO_2_ seeps provide one of the best opportunities for assessing the long-term impact of OA on marine holobionts *in situ*. The pH levels experienced by the sampled corals at the volcanic seep in the D’Entrecasteaux Islands varied from 7.44 to 7.85, indicating that some of these corals experienced harsher OA conditions than those predicted for the next 100 years. This strengthens our findings that *p*CO_2_-tolerant corals are accompanied by a robust bacterial community, similar to what was found in their endolithic community (Marcelino et al., 2017). Coral-associated bacterial communities were stable across CO_2_ seep and control sites, contrasting with the shift previously observed in corals that were more *p*CO_2_-sensitive (Morrow et al., 2014). Instead, bacterial communities were mainly influenced by geographic site differences. These data, combined with results from previous studies of massive *Porites* at the PNG seeps, suggest that massive *Porites* are able to maintain and regulate internal pH in low pH/high *p*CO_2_ environments and subsequently provide a stable habitat for microbial partners. This potentially leads to a positive feedback for the resilience of this species at low pH, thereby contributing to its potential dominance and success in future OA scenarios.

## Data Availability Statement

The datasets generated for this study can be found in the NCBI Sequence Read Archive via accession number SRP148584.

## Ethics Statement

This study was exempt from these requirements as research involving corals is not required to obtain ethics approval.

## Author Contributions

DB, KM, PO, BW, and KF conceived, designed, and coordinated the study. KM collected specimens and field data. PO and KM carried out molecular lab work and photo analysis, SF carried out ^14^C lab work and analysis. HS completed molecular and statistical analysis. PO drafted the manuscript with input from all authors. All authors gave final approval for publication.

## Conflict of Interest Statement

The authors declare that the research was conducted in the absence of any commercial or financial relationships that could be construed as a potential conflict of interest.
