# Supplementary material for: Elevated CO2 Has Little Influence on the Bacterial Communities Associated With the pH-Tolerant Coral, Massive Porites spp
Source: Front Microbiol. 2018 Nov 1;9:2621. doi: 10.3389/fmicb.2018.02621 (PMC6221987; doi:10.3389/fmicb.2018.02621)
Supplement: Supplementary file 2 [file Table_2.DOCX]

Supplementary Material

**Elevated CO_2_ Has Little Influence on the Bacterial Communities Associated with the pH-tolerant Coral, Massive *Porites spp.***

**Paul A. O’Brien, Hillary A. Smith, Stewart Fallon, Katharina Fabricius, Bette L. Willis, Kathleen M. Morrow, David G. Bourne***

*** Correspondence:** Corresponding Author: david.bourne@jcu.edu.au


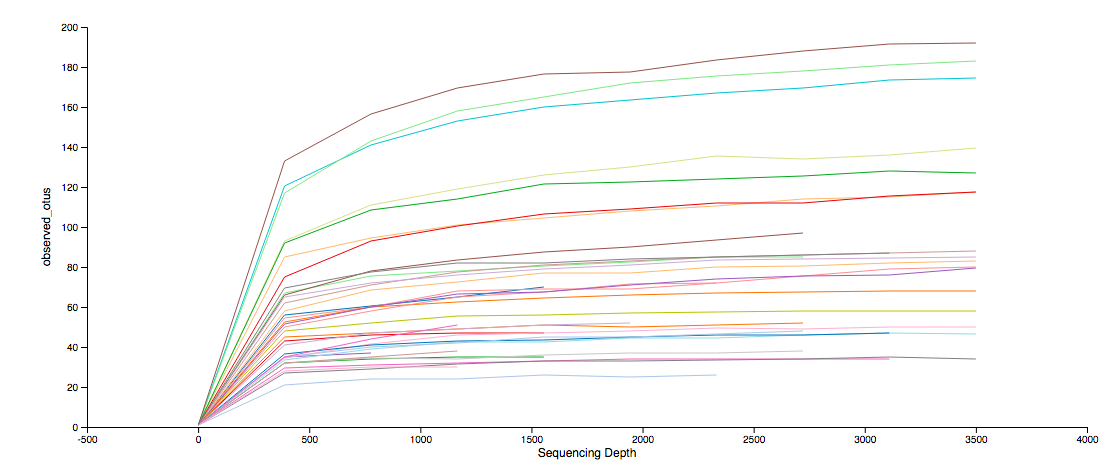


**Supplementary Figure S1.** Alpha rarefaction plot demonstrating the number of detected OTUs with sequencing depth.

*
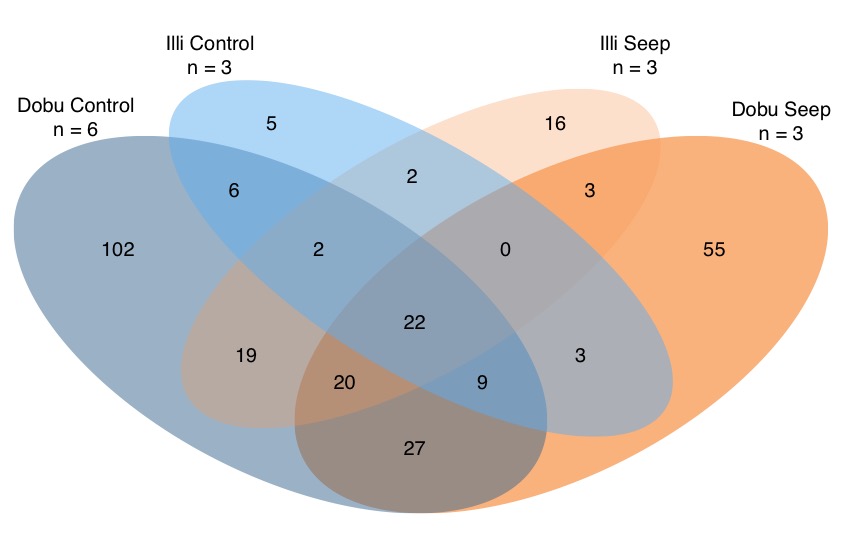
*

**Supplementary Figure S2.** Venn diagram showing the number of genera distinct to each grouping. Twenty-two taxa were found in 100% of samples.

**Supplementary Figure S3**. Percent cover of all substrata with >5% cover.

**Supplementary Equations.**

A simple two component mixing model can be used to determine the amount of additional ^14^C free CO_2_ that was added to seep site corals:

$${F^{14}C}_{seep coral}= f_{contam}* {F^{14}C}_{contamin}+\left( 1-f_{contam} \right)* {F^{14}C}_{control coral}$$

As the seep CO_2_ contains virtually no radiocarbon, the value of F^14^C_contam_ is equal to zero. Therefore, the equation becomes:

$${F^{14}C}_{seep coral}=\left( 1-f_{contam} \right)* {F^{14}C}_{control coral}$$

which can be rearranged to give f_contam_:

$$f_{contam}=\left( {F^{14}C}_{seep coral}-{F^{14}C}_{control coral} \right)/ {F^{14}C}_{control coral}$$

f_contam_ was used to calculate the DIC of the seep coral from the control coral DIC. The DIC of the seep site is equal to the DIC of the control site plus the amount of additional DIC from the seep required to dilute the control F^14^C to the impacted F^14^C value:

$${DIC}_{control}= {(1-f}_{contam})* {DIC}_{seep}$$

$${DIC}_{seep}= \frac{{DIC}_{control}}{{(1-f}_{contam})}$$

DIC values for the impacted sites were calculated using *in situ* DIC measurements from the control coral sites [37] and f_contam_ values calculated from the difference between control and impacted F^14^C.

In order to model seawater pH, a comparison between the F^14^C from the control site corals and the seep site corals was made. Since the seep CO_2_ does not contain ^14^C the extra carbon (CO_2_) from the seeps will cause the seep corals to have a lower F^14^C than the control site corals. A simple two component mixing model can be used to determine the amount of additional ^14^C free CO_2_ that was added to seep site corals.

**Supplementary Table S1**. ^14^C Analysis calculated from the skeletons of *Porites* samples collected at both seep and control sites. As control ^14^C and control pH is needed to calculate seep pH, only seep values are given (see methods). f contam refers to the difference in F^14^C between control and seep samples.

| *Sample* | ***F ^14^C*** | ***f contam*** | ***1-f contam*** | ***pH*** | ***pCO_2_*** | ***CO2 in (mmol/kgSW)*** | ***Ω Ar*** |
| --- | --- | --- | --- | --- | --- | --- | --- |
| Dobu Ctrl | 1.0513 |  |  |  |  |  |  |
| Dobu Ctrl | 1.0540 |  |  |  |  |  |  |
| Dobu Ctrl | 1.0502 |  |  |  |  |  |  |
| Dobu Ctrl 2 | 1.0439 |  |  |  |  |  |  |
| Dobu Ctrl 2 | 1.0432 |  |  |  |  |  |  |
| Dobu Ctrl 2 | 1.0435 |  |  |  |  |  |  |
| Dobu Seep | 0.9729 | 0.075 | 0.925 | 7.67 | 1089.9 | 1093.2 | 1.90 |
| Dobu Seep | 0.9717 | 0.076 | 0.924 | 7.67 | 1108.2 | 1111.6 | 1.87 |
| Dobu Seep | 0.9948 | 0.054 | 0.946 | 7.78 | 818.6 | 821.1 | 2.35 |
| Illi Ctrl | 1.0566 |  |  |  |  |  |  |
| Illi Ctrl | 1.0473 |  |  |  |  |  |  |
| Illi Ctrl | 1.0507 |  |  |  |  |  |  |
| Illi Seep | 0.9264 | 0.119 | 0.881 | 7.44 | 1960.4 | 1966.5 | 1.12 |
| Illi Seep | 0.9991 | 0.050 | 0.950 | 7.85 | 685.8 | 688.0 | 2.59 |
| Illi Seep | 0.9293 | 0.116 | 0.884 | 7.46 | 1864.9 | 1870.7 | 1.17 |
