# Supplementary material for: Elevated CO2 Has Little Influence on the Bacterial Communities Associated With the pH-Tolerant Coral, Massive Porites spp
Source: Front Microbiol. 2018 Nov 1;9:2621. doi: 10.3389/fmicb.2018.02621 (PMC6221987; doi:10.3389/fmicb.2018.02621)
Supplement: Supplementary file 3 [file Table_3.docx]

Supplementary Table S2. Summary statistics for relative abundance of all major bacteria classifications presented in results^[[1]](#footnote-1)^

|  | **n** | **mean** | **median** | **min** | **max** | **range** | **sd** | **se** |
| --- | --- | --- | --- | --- | --- | --- | --- | --- |
| **Dobu Control** |  |  |  |  |  |  |  |  |
| **(p) Proteobacteria** | **6** | **56.9** | **60.7** | **29.2** | **82.0** | **52.8** | **23.4** | **9.6** |
| (c) *Alphaproteobacteria* | 6 | 17.0 | 15.3 | 10.5 | 24.5 | 14.0 | 5.6 | 2.3 |
| (g) *Maritimibacter* | 6 | 0.9 | 1.1 | 0.0 | 1.5 | 1.5 | 0.7 | 0.3 |
| (g) *Thalassospira* | 6 | 1.0 | 0.8 | 0.4 | 1.8 | 1.4 | 0.5 | 0.2 |
| (g) *Rhodospirillaceae* uncultured | 6 | 0.9 | 0.5 | 0.0 | 2.5 | 2.5 | 1.0 | 0.4 |
| (g) *Maricaulis* | 6 | 0.8 | 0.7 | 0.5 | 1.0 | 0.6 | 0.2 | 0.1 |
| (g) *Ruegeria* | 6 | 1.7 | 1.3 | 0.7 | 3.1 | 2.4 | 1.0 | 0.4 |
| (f) *Phyllobacteriaceae* | 6 | 0.6 | 0.7 | 0.0 | 1.3 | 1.3 | 0.5 | 0.2 |
| (c) *Deltaproteobacteria* | 6 | 1.5 | 1.7 | 0.0 | 2.6 | 2.6 | 1.0 | 0.4 |
| (c) *Epsilonproteobacteria* | 6 | 0.5 | 0.0 | 0.0 | 2.4 | 2.4 | 1.0 | 0.4 |
| (c) *Betaproteobacteria* | 6 | 0.0 | 0.0 | 0.0 | 0.0 | 0.0 | 0.0 | 0.0 |
| (c) *Gammaproteobacteria* | 6 | 37.3 | 31.7 | 10.5 | 70.8 | 60.4 | 26.2 | 10.7 |
| (g) *Endozoicomonas* | 6 | 10.1 | 2.7 | 1.3 | 48.5 | 47.2 | 18.8 | 7.7 |
| (g) *Vibrio* | 6 | 2.1 | 2.1 | 1.5 | 2.6 | 1.1 | 0.5 | 0.2 |
| (g) *Caedibacter* | 6 | 0.4 | 0.2 | 0.0 | 1.7 | 1.7 | 0.7 | 0.3 |
| (o) *Alteromonadales* | 6 | 13.4 | 10.4 | 3.0 | 35.6 | 32.6 | 11.8 | 4.8 |
| (g) *Alteromonas* | 6 | 1.5 | 1.5 | 0.3 | 2.8 | 2.4 | 0.8 | 0.3 |
| (g) *Pseudoalteromonas* | 6 | 0.8 | 0.4 | 0.0 | 3.5 | 3.5 | 1.3 | 0.5 |
| (g) Marinobacter | 6 | 10.4 | 4.5 | 1.9 | 33.3 | 31.4 | 12.1 | 4.9 |
| **(p) Bacteroidetes** | **6** | **14.0** | **13.7** | **5.3** | **25.8** | **20.5** | **7.5** | **3.1** |
| (c) *Flavobacteriia* | 6 | 10.6 | 10.6 | 3.6 | 19.6 | 16.0 | 5.5 | 2.2 |
| (f) *Flavobacteriaceae* | 6 | 8.1 | 8.6 | 1.7 | 15.5 | 13.8 | 4.6 | 1.9 |
| (g) *Muricauda* | 6 | 1.6 | 1.1 | 0.0 | 3.3 | 3.3 | 1.3 | 0.5 |
| (g) *Winogradskyella* | 6 | 1.7 | 1.5 | 1.0 | 3.1 | 2.1 | 0.8 | 0.3 |
| (c) *Cytophagia* | 6 | 2.0 | 1.3 | 0.0 | 4.9 | 4.9 | 1.8 | 0.8 |
| **(p) Verrucomicrobia** | **6** | **1.4** | **0.8** | **0.0** | **3.5** | **3.5** | **1.6** | **0.7** |
| **(p) Acidobacteria** | **6** | **0.4** | **0.0** | **0.0** | **1.5** | **1.5** | **0.6** | **0.3** |
| **(p) Actinobacteria** | **6** | **0.7** | **0.6** | **0.0** | **1.7** | **1.7** | **0.6** | **0.3** |
| (f) *OM1 clade* | 6 | 0.2 | 0.2 | 0.0 | 0.5 | 0.5 | 0.2 | 0.1 |
| **(p) Chlamydiae** | **6** | **0.4** | **0.4** | **0.0** | **1.0** | **1.0** | **0.4** | **0.2** |
| (g) *Simkaniaceae uncultured* | 6 | 0.2 | 0.2 | 0.0 | 0.5 | 0.5 | 0.2 | 0.1 |
| **(p) Chlorobi** | **6** | **16.2** | **3.0** | **0.0** | **46.0** | **46.0** | **22.7** | **9.3** |
| (g) *Prosthecochloris* | 6 | 15.5 | 2.7 | 0.0 | 43.8 | 43.8 | 21.8 | 8.9 |
| **(p) Chloroflexi** | **6** | **0.1** | **0.1** | **0.0** | **0.3** | **0.3** | **0.2** | **0.1** |
| **(p) Cyanobacteria** | **6** | **5.2** | **5.0** | **1.3** | **8.8** | **7.5** | **2.5** | **1.0** |
| (g) *Rivularia* | 6 | 1.1 | 1.0 | 0.7 | 1.5 | 0.8 | 0.3 | 0.1 |
| (g) *Synechococcus* | 6 | 0.0 | 0.0 | 0.0 | 0.1 | 0.1 | 0.0 | 0.0 |
| **(p) Parcubacteria** | **6** | **0.8** | **0.8** | **0.0** | **1.4** | **1.4** | **0.5** | **0.2** |
| **OtherBacteria** | **6** | **0.7** | **0.7** | **0.0** | **1.4** | **1.4** | **0.5** | **0.2** |
|  |  |  |  |  |  |  |  |  |
|  | **n** | **mean** | **median** | **min** | **max** | **range** | **sd** | **se** |
| **Dobu Seep** |  |  |  |  |  |  |  |  |
| **(p) Proteobacteria** | **3** | **61.6** | **70.2** | **44.2** | **70.5** | **26.3** | **15.1** | **8.7** |
| (c) *Alphaproteobacteria* | 3 | 20.4 | 18.1 | 16.7 | 26.3 | 9.5 | 5.1 | 3.0 |
| (g) *Maritimibacter* | 3 | 0.4 | 0.0 | 0.0 | 1.3 | 1.3 | 0.8 | 0.4 |
| (g) *Thalassospira* | 3 | 1.1 | 0.9 | 0.8 | 1.6 | 0.8 | 0.4 | 0.3 |
| (g) *Rhodospirillaceae* uncultured | 3 | 1.1 | 1.2 | 0.6 | 1.4 | 0.9 | 0.4 | 0.3 |
| (g) *Maricaulis* | 3 | 1.6 | 1.5 | 1.1 | 2.2 | 1.2 | 0.6 | 0.3 |
| (g) *Ruegeria* | 3 | 3.3 | 3.5 | 0.0 | 6.4 | 6.4 | 3.2 | 1.9 |
| (f) *Phyllobacteriaceae* | 3 | 0.3 | 0.0 | 0.0 | 0.8 | 0.8 | 0.5 | 0.3 |
| (c) *Betaproteobacteria* | 3 | 0.1 | 0.0 | 0.0 | 0.2 | 0.2 | 0.1 | 0.1 |
| (c) *Deltaproteobacteria* | 3 | 1.4 | 1.1 | 0.9 | 2.2 | 1.3 | 0.7 | 0.4 |
| (c) *Epsilonproteobacteria* | 3 | 0.4 | 0.0 | 0.0 | 1.2 | 1.2 | 0.7 | 0.4 |
| (c) *Gammaproteobacteria* | 3 | 37.3 | 36.0 | 26.0 | 49.8 | 23.8 | 12.0 | 6.9 |
| (g) *Endozoicomonas* | 3 | 10.4 | 11.8 | 7.6 | 11.9 | 4.4 | 2.5 | 1.4 |
| (g) *Vibrio* | 3 | 5.0 | 6.2 | 2.1 | 6.7 | 4.6 | 2.5 | 1.4 |
| (g) *Caedibacter* | 3 | 0.1 | 0.0 | 0.0 | 0.3 | 0.3 | 0.2 | 0.1 |
| (o) *Alteromonadales* | 3 | 11.6 | 13.6 | 3.5 | 17.6 | 14.0 | 7.2 | 4.2 |
| (g) *Alteromonas* | 3 | 1.9 | 2.3 | 0.5 | 2.8 | 2.3 | 1.2 | 0.7 |
| (g) *Pseudoalteromonas* | 3 | 3.4 | 3.3 | 1.7 | 5.2 | 3.5 | 1.7 | 1.0 |
| (g) Marinobacter | 3 | 5.4 | 7.2 | 1.3 | 7.7 | 6.3 | 3.5 | 2.0 |
| **(p) Bacteroidetes** | **3** | **11.8** | **11.4** | **10.0** | **13.9** | **3.9** | **2.0** | **1.1** |
| (c) *Flavobacteriia* | 3 | 9.1 | 9.1 | 7.6 | 10.6 | 3.0 | 1.5 | 0.9 |
| (f) *Flavobacteriaceae* | 3 | 7.4 | 8.7 | 4.8 | 8.7 | 3.9 | 2.2 | 1.3 |
| (g) *Muricauda* | 3 | 0.6 | 0.6 | 0.0 | 1.2 | 1.2 | 0.6 | 0.3 |
| (g) *Winogradskyella* | 3 | 2.5 | 1.3 | 1.3 | 4.9 | 3.6 | 2.1 | 1.2 |
| (c) *Cytophagia* | 3 | 1.7 | 0.3 | 0.0 | 4.9 | 4.9 | 2.7 | 1.6 |
| **(p) Verrucomicrobia** | **3** | **1.9** | **0.0** | **0.0** | **5.6** | **5.6** | **3.2** | **1.9** |
| **(p) Acidobacteria** | **3** | **2.0** | **2.7** | **0.6** | **2.8** | **2.2** | **1.3** | **0.7** |
| **(p) Actinobacteria** | **3** | **4.6** | **3.0** | **1.2** | **9.6** | **8.5** | **4.5** | **2.6** |
| (f) *OM1 clade* | 3 | 1.4 | 0.9 | 0.8 | 2.5 | 1.7 | 0.9 | 0.5 |
| **(p) Chlamydiae** | **3** | **1.5** | **0.8** | **0.6** | **3.1** | **2.4** | **1.4** | **0.8** |
| (g) *Simkaniaceae uncultured* | 3 | 0.4 | 0.6 | 0.0 | 0.7 | 0.7 | 0.4 | 0.2 |
| **(p) Chlorobi** | **3** | **0.3** | **0.0** | **0.0** | **1.0** | **1.0** | **0.6** | **0.3** |
| (g) *Prosthecochloris* | 3 | 0.3 | 0.0 | 0.0 | 0.9 | 0.9 | 0.5 | 0.3 |
| **(p) Chloroflexi** | **3** | **2.3** | **2.2** | **0.0** | **4.5** | **4.5** | **2.3** | **1.3** |
| **(p) Cyanobacteria** | **3** | **2.7** | **1.4** | **0.6** | **6.2** | **5.6** | **3.1** | **1.8** |
| (g) *Rivularia* | 3 | 0.5 | 0.0 | 0.0 | 1.5 | 1.5 | 0.9 | 0.5 |
| (g) *Synechococcus* | 3 | 0.2 | 0.0 | 0.0 | 0.5 | 0.5 | 0.3 | 0.2 |
| **(p) Parcubacteria** | **3** | **2.0** | **2.6** | **0.0** | **3.5** | **3.5** | **1.8** | **1.0** |
| **OtherBacteria** | **3** | **2.8** | **1.2** | **0.3** | **6.9** | **6.6** | **3.6** | **2.1** |
|  |  |  |  |  |  |  |  |  |
|  | **n** | **mean** | **median** | **min** | **max** | **range** | **sd** | **se** |
| **Illi Control** |  |  |  |  |  |  |  |  |
| **(p) Proteobacteria** | **3** | **94.7** | **94.8** | **93.6** | **95.7** | **2.1** | **1.1** | **0.6** |
| (c) *Alphaproteobacteria* | 3 | 5.6 | 5.9 | 4.7 | 6.2 | 1.5 | 0.8 | 0.5 |
| (g) *Maritimibacter* | 3 | 1.0 | 0.8 | 0.8 | 1.6 | 0.8 | 0.5 | 0.3 |
| (g) *Thalassospira* | 3 | 0.6 | 0.6 | 0.3 | 0.9 | 0.6 | 0.3 | 0.2 |
| (g) *Rhodospirillaceae* uncultured | 3 | 0.2 | 0.0 | 0.0 | 0.7 | 0.7 | 0.4 | 0.2 |
| (g) *Maricaulis* | 3 | 0.7 | 0.4 | 0.0 | 1.7 | 1.7 | 0.9 | 0.5 |
| (g) *Ruegeria* | 3 | 1.6 | 1.5 | 1.4 | 1.8 | 0.4 | 0.2 | 0.1 |
| (f) *Phyllobacteriaceae* | 3 | 0.4 | 0.5 | 0.0 | 0.9 | 0.9 | 0.4 | 0.3 |
| (c) *Betaproteobacteria* | 3 | 0.0 | 0.0 | 0.0 | 0.0 | 0.0 | 0.0 | 0.0 |
| (c) *Deltaproteobacteria* | 3 | 0.6 | 0.6 | 0.2 | 0.9 | 0.7 | 0.4 | 0.2 |
| (c) *Epsilonproteobacteria* | 3 | 0.0 | 0.0 | 0.0 | 0.0 | 0.0 | 0.0 | 0.0 |
| (c) *Gammaproteobacteria* | 3 | 88.4 | 88.1 | 86.7 | 90.6 | 3.9 | 2.0 | 1.1 |
| (g) *Endozoicomonas* | 3 | 79.6 | 80.1 | 74.4 | 84.2 | 9.9 | 5.0 | 2.9 |
| (g) *Vibrio* | 3 | 1.9 | 1.3 | 0.5 | 3.8 | 3.3 | 1.7 | 1.0 |
| (g) *Caedibacter* | 3 | 0.3 | 0.2 | 0.2 | 0.3 | 0.1 | 0.0 | 0.0 |
| (o) *Alteromonadales* | 3 | 2.5 | 2.6 | 2.1 | 2.7 | 0.6 | 0.3 | 0.2 |
| (g) *Alteromonas* | 3 | 0.9 | 0.7 | 0.5 | 1.3 | 0.8 | 0.4 | 0.2 |
| (g) *Pseudoalteromonas* | 3 | 0.5 | 0.5 | 0.4 | 0.6 | 0.2 | 0.1 | 0.1 |
| (g) Marinobacter | 3 | 0.9 | 0.9 | 0.8 | 1.0 | 0.2 | 0.1 | 0.1 |
| **(p) Bacteroidetes** | **3** | **2.7** | **2.6** | **2.1** | **3.4** | **1.3** | **0.6** | **0.4** |
| (c) *Flavobacteriia* | 3 | 2.0 | 1.8 | 1.4 | 2.9 | 1.5 | 0.8 | 0.4 |
| (f) *Flavobacteriaceae* | 3 | 2.0 | 1.8 | 1.4 | 2.8 | 1.4 | 0.7 | 0.4 |
| (g) *Muricauda* | 3 | 0.6 | 0.6 | 0.5 | 0.7 | 0.2 | 0.1 | 0.0 |
| (g) *Winogradskyella* | 3 | 1.2 | 1.1 | 0.9 | 1.5 | 0.6 | 0.3 | 0.2 |
| (c) *Cytophagia* | 3 | 0.1 | 0.0 | 0.0 | 0.4 | 0.4 | 0.2 | 0.1 |
| **(p) Verrucomicrobia** | **3** | **0.0** | **0.0** | **0.0** | **0.0** | **0.0** | **0.0** | **0.0** |
| **(p) Acidobacteria** | **3** | **0.0** | **0.0** | **0.0** | **0.0** | **0.0** | **0.0** | **0.0** |
| **(p) Actinobacteria** | **3** | **0.0** | **0.0** | **0.0** | **0.0** | **0.0** | **0.0** | **0.0** |
| (f) *OM1 clade* | 3 | 0.0 | 0.0 | 0.0 | 0.0 | 0.0 | 0.0 | 0.0 |
| **(p) Chlamydiae** | **3** | **0.4** | **0.3** | **0.3** | **0.4** | **0.1** | **0.1** | **0.0** |
| (g) *Simkaniaceae uncultured* | 3 | 0.3 | 0.3 | 0.3 | 0.4 | 0.1 | 0.0 | 0.0 |
| **(p) Chlorobi** | **3** | **0.9** | **0.8** | **0.8** | **0.9** | **0.1** | **0.1** | **0.0** |
| (g) *Prosthecochloris* | 3 | 0.8 | 0.8 | 0.8 | 0.9 | 0.1 | 0.0 | 0.0 |
| **(p) Chloroflexi** | **3** | **0.0** | **0.0** | **0.0** | **0.0** | **0.0** | **0.0** | **0.0** |
| **(p) Cyanobacteria** | **3** | **0.8** | **0.7** | **0.4** | **1.3** | **0.9** | **0.4** | **0.3** |
| (g) *Rivularia* | 3 | 0.4 | 0.5 | 0.0 | 0.5 | 0.5 | 0.3 | 0.2 |
| (g) *Synechococcus* | 3 | 0.2 | 0.2 | 0.0 | 0.3 | 0.3 | 0.2 | 0.1 |
| **(p) Parcubacteria** | **3** | **0.0** | **0.0** | **0.0** | **0.0** | **0.0** | **0.0** | **0.0** |
| **OtherBacteria** | **3** | **0.0** | **0.0** | **0.0** | **0.0** | **0.0** | **0.0** | **0.0** |
|  |  |  |  |  |  |  |  |  |
|  | **n** | **mean** | **median** | **min** | **max** | **range** | **sd** | **se** |
| **Illi Seep** |  |  |  |  |  |  |  |  |
| **(p) Proteobacteria** | **3** | **75.0** | **73.3** | **62.1** | **89.4** | **27.3** | **13.7** | **7.9** |
| (c) *Alphaproteobacteria* | 3 | 18.3 | 18.7 | 17.5 | 18.7 | 1.2 | 0.7 | 0.4 |
| (g) *Maritimibacter* | 3 | 3.1 | 2.4 | 1.4 | 5.5 | 4.0 | 2.1 | 1.2 |
| (g) *Thalassospira* | 3 | 1.9 | 1.9 | 0.8 | 3.0 | 2.2 | 1.1 | 0.6 |
| (g) *Rhodospirillaceae* uncultured | 3 | 0.7 | 0.9 | 0.0 | 1.2 | 1.2 | 0.6 | 0.4 |
| (g) *Maricaulis* | 3 | 0.8 | 0.9 | 0.0 | 1.5 | 1.5 | 0.8 | 0.4 |
| (g) *Ruegeria* | 3 | 2.9 | 3.2 | 1.9 | 3.6 | 1.7 | 0.9 | 0.5 |
| (f) *Phyllobacteriaceae* | 3 | 1.4 | 1.9 | 0.0 | 2.4 | 2.4 | 1.3 | 0.7 |
| (c) *Betaproteobacteria* | 3 | 0.0 | 0.0 | 0.0 | 0.0 | 0.0 | 0.0 | 0.0 |
| (c) *Deltaproteobacteria* | 3 | 0.8 | 0.9 | 0.0 | 1.4 | 1.4 | 0.7 | 0.4 |
| (c) *Epsilonproteobacteria* | 3 | 0.2 | 0.0 | 0.0 | 0.6 | 0.6 | 0.3 | 0.2 |
| (c) *Gammaproteobacteria* | 3 | 55.5 | 53.7 | 42.6 | 70.1 | 27.5 | 13.9 | 8.0 |
| (g) *Endozoicomonas* | 3 | 38.3 | 33.2 | 23.8 | 58.1 | 34.3 | 17.7 | 10.2 |
| (g) *Vibrio* | 3 | 4.5 | 3.8 | 0.0 | 9.7 | 9.7 | 4.9 | 2.8 |
| (g) *Caedibacter* | 3 | 0.8 | 0.6 | 0.2 | 1.5 | 1.3 | 0.7 | 0.4 |
| (o) *Alteromonadales* | 3 | 5.2 | 6.3 | 2.8 | 6.4 | 3.5 | 2.0 | 1.2 |
| (g) *Alteromonas* | 3 | 1.1 | 0.0 | 0.0 | 3.4 | 3.4 | 2.0 | 1.1 |
| (g) *Pseudoalteromonas* | 3 | 0.6 | 0.7 | 0.0 | 1.0 | 1.0 | 0.5 | 0.3 |
| (g) Marinobacter | 3 | 3.0 | 1.9 | 1.7 | 5.6 | 3.9 | 2.2 | 1.3 |
| **(p) Bacteroidetes** | **3** | **15.8** | **11.7** | **7.7** | **28.0** | **20.3** | **10.7** | **6.2** |
| (c) *Flavobacteriia* | 3 | 13.4 | 10.7 | 7.7 | 21.9 | 14.1 | 7.5 | 4.3 |
| (f) *Flavobacteriaceae* | 3 | 9.9 | 8.9 | 4.5 | 16.3 | 11.8 | 6.0 | 3.5 |
| (g) *Muricauda* | 3 | 1.5 | 1.3 | 0.0 | 3.2 | 3.2 | 1.6 | 0.9 |
| (g) *Winogradskyella* | 3 | 4.0 | 4.1 | 3.5 | 4.4 | 0.9 | 0.4 | 0.3 |
| (c) *Cytophagia* | 3 | 1.7 | 0.5 | 0.0 | 4.5 | 4.5 | 2.5 | 1.4 |
| **(p) Verrucomicrobia** | **3** | **0.6** | **0.8** | **0.0** | **0.9** | **0.9** | **0.5** | **0.3** |
| **(p) Acidobacteria** | **3** | **0.1** | **0.0** | **0.0** | **0.3** | **0.3** | **0.1** | **0.1** |
| **(p) Actinobacteria** | **3** | **2.5** | **2.3** | **0.0** | **5.1** | **5.1** | **2.6** | **1.5** |
| (f) *OM1 clade* | 3 | 0.0 | 0.0 | 0.0 | 0.0 | 0.0 | 0.0 | 0.0 |
| **(p) Chlamydiae** | **3** | **1.4** | **1.8** | **0.5** | **2.0** | **1.5** | **0.8** | **0.5** |
| (g) *Simkaniaceae uncultured* | 3 | 0.2 | 0.0 | 0.0 | 0.5 | 0.5 | 0.3 | 0.2 |
| **(p) Chlorobi** | **3** | **1.2** | **1.3** | **1.1** | **1.3** | **0.2** | **0.1** | **0.1** |
| (g) *Prosthecochloris* | 3 | 1.2 | 1.2 | 1.1 | 1.2 | 0.2 | 0.1 | 0.0 |
| **(p) Chloroflexi** | **3** | **0.9** | **0.0** | **0.0** | **2.6** | **2.6** | **1.5** | **0.9** |
| **(p) Cyanobacteria** | **3** | **1.1** | **0.9** | **0.0** | **2.2** | **2.2** | **1.1** | **0.7** |
| (g) *Rivularia* | 3 | 0.3 | 0.0 | 0.0 | 0.8 | 0.8 | 0.5 | 0.3 |
| (g) *Synechococcus* | 3 | 0.3 | 0.4 | 0.0 | 0.4 | 0.4 | 0.2 | 0.1 |
| **(p) Parcubacteria** | **3** | **0.0** | **0.0** | **0.0** | **0.0** | **0.0** | **0.0** | **0.0** |
| **OtherBacteria** | **3** | **0.4** | **0.5** | **0.0** | **0.7** | **0.7** | **0.4** | **0.2** |

1. n = sample size, min = minimum relative abundance, max = maximum relative abundance, sd = standard deviation, se = standard error.

   p = phylum, c = class, o = order, f = family, g = genus [↑](#footnote-ref-1)
